# Mr. Martin, on the Treatment of Burns and Scalds

**Published:** 1808-01

**Authors:** Peter Martin

**Affiliations:** the P.G.Y.C. Dunning


### *To the Editors of the Medical and Phyfical Journal.*

GENTLEMEN,

HAVING had a considerable share of experience in the treatment of burns and scalds, I solicit leave, through the medium of your valuable publication, to offer a few remarks on the aqueous and terebinthinate plans of cure, which have, of late, been the subject of so much controversial investigation between Drs. Kentish and Kinglake.

I have tried both modes of treatment, and could have forwarded a long and accurate detail of numerous cases on each side of the question; but as their insertion at full length would have occupied, perhaps, a whole Number of your Journal, it occurs to me that the following short epitome of the result of these experiments, is all the useful practical conclusions which could have been drawn from their perusal.

Dr. Kinglake's topical refrigeration is, in general, a judicious application in the first stage of scalds; and has, on many occasions, appeared to work wonders in my hands, especially when the scalded parts were so situated, as to admit of full and continued immersion in the cooling liquid. Few patients, however, will be found to put confidence in cold water, as a medicine or remedy, in any disorder; and, upon the whole, it is not a scientific application.

Dr. Kinglake should revolutionize his formula, before a more intimate acquaintance with the populace show him the folly of redundant simplicity, and convince him of the necessity of substituting, at least apparently, a different remedy. What I used was a solution of acetis plumbi, or sometimes oxide of hydrogen, made of the colour of brandy, with hæmatoxylum campeachianum (for I durst not mention or prescribe water); but the former always answered best, when about the strength of three drachms to an English gallon.

That a weak solution of lead is a proper application to scalds, where the inflammatory symptoms run high, is a medical fact, which no practitioner, I presume, will deny; but that it should be used in every injury of the kind, is a proposition, which every intelligent, well experienced practitioner will be disposed to negative.

Burns and scalds have generally been treated of under one head, in a conjunct kind of manner. They are injuries inflicted by the contact of some body, either fluid or solid, possessing such an excess of caloric, as either vesicates, inflames, or destroys, the surface of the parts touched; and are at times very different, not only in degree, but more especially in their nature, requiring occasionally opposite modes of treatment.

In extensive burns (occasioned by hot iron, live coals, explosions of gunpowder, hydrogenous gas, &c. or by the clothes taking fire), it sometimes happens, that instead of active inflammation being excited, Nature flags at once, and the parts injured put on an appearance resembling gangrene. It is under these, and analogous circumstances, that the application of ol. terebinth. has been most beneficial; and, in such cases, no remedy appears to answer better, as far as my experience at present goes; but in other cases of lesser burns, the patients complained of additional pain from this application, and would not persist in its use, but laid it aside for the old but common application of linseed oil, which, they said, gave much more ease. However, beyond all doubt, the oil of turpentine is a valuable remedy in many cases of burns; but its use, like the aqueous plan of treatment, is only judicious under certain circumstances, and with due limitations; for neither the one nor the other, though proper at first, ought to be continued during the whole course of the cure, as the exposed parts, after the healing process is instituted, is best defended from irritation by the application of some mild ointment, varied as the state of the parts seem to indicate.

But to conclude, it will now and then happen, both in burns and scalds, that after one of the above plans first had recourse to, whether aqueous or terebinthinate, has proved inefficient, or perhaps injurious, the application of the other will succeed to our wishes; and, in other cases, the application of linniment. aqua calcis has proved preferable to either, especially in irritable nervous habits. But in all cases of severe burns and scalds, whatever may be the external mode of treatment, the judicious use of proper internals will always be found greatly to expedite and facilitate the cure.

It would be acceptable to many of your readers, if these disputants would close this controversy; for both modes of cure are occasionally successful, and neither the one nor the other can be banished from practice.

With all due deference to Drs. Kentish and Kinglake, who, for their zealous exertions towards improvement, are well well deserving of the applause of all the lovers of literature, I am, Gentlemen, your most obedient humble Servant,

PETER MARTIN, of the P.G.Y.C.

